# Clinical challenges and individualized management of hypertensive emergency complicated by acute ischemic stroke: A case report and literature review

**DOI:** 10.1097/MD.0000000000050070

**Published:** 2026-09-04

**Authors:** Weihao Wang, Don Zhu, Xiaoyu Liu, Keyuan Zhao, Xiaoyang Zhang, Zhouyang Gu

**Affiliations:** a Department of Emergency Medicine, Shaoxing Central Hospital, Shaoxing, Zhejiang, China; b Department of Urology, Shaoxing People’s Hospital, Shaoxing, Zhejiang, China.

**Keywords:** acute ischemic stroke, alteplase, blood pressure management, hypertensive emergency, mechanical thrombectomy

## Abstract

**Rationale:**

Hypertensive emergency complicated by acute ischemic stroke (AIS) represents a life-threatening clinical condition. Abrupt elevations in blood pressure not only increase the risk of stroke-related complications but also predispose patients to cerebral perfusion imbalance and target-organ injury. However, there is no consensus regarding the optimal antihypertensive strategy or individualized management protocol in this setting.

**Patient concerns:**

Three individuals with preexisting chronic hypertension developed acute ischemic stroke in the context of a hypertensive emergency, posing challenges in evaluating disease evolution, optimizing individualized treatment strategies, and assessing long-term prognosis.

**Diagnoses:**

Based on clinical manifestations and imaging examinations, all 3 cases were diagnosed as hypertensive emergencies with concomitant AIS.

**Interventions:**

In the emergency department, all patients were managed with urapidil administered through a continuous microinfusion pump to achieve blood pressure control. Following stabilization, intravenous thrombolysis was performed in case 1, whereas mechanical thrombectomy was undertaken in cases 2 and 3.

**Outcomes:**

After therapeutic intervention, all patients achieved blood pressure stabilization within the target range and demonstrated significant neurological recovery in the acute phase. Cases 1 and 2 demonstrated favorable clinical recovery without major complications during follow-up. Case 3 underwent successful emergency thrombectomy but was transferred to another institution shortly thereafter and was subsequently lost to follow-up.

**Lessons:**

Treating hypertensive emergency in the setting of AIS demands careful appraisal of the patient’s condition, hemodynamic profile, and cerebral perfusion needs to guide individualized therapy. Effective yet safe blood pressure reduction is critical to avoid cerebral hypoperfusion. Long-term prognosis can be further optimized through a multidisciplinary team (MDT), close follow-up, strict medication compliance, and targeted lifestyle interventions.

## 1. Introduction

Hypertensive emergency is defined as a critical clinical condition characterized by a rapid and severe elevation in blood pressure (typically systolic blood pressure (SBP) >180 mm Hg and/or diastolic blood pressure >120 mm Hg), leading to progressive injury of target organs such as the brain, heart, and kidneys.^[1]^ According to current international consensus, such as the 2020 International Society of Hypertension Global Hypertension Practice Guidelines, patients with hypertensive emergency should undergo a gradual reduction in blood pressure by 20% to 25% within the first 1 to 2 hours in order to prevent progressive target-organ damage.^[2]^ This condition is associated with high rates of mortality and disability, and is particularly prevalent among patients with poorly controlled chronic hypertension or those who discontinue antihypertensive medications without medical supervision. Among the target-organ damages caused by hypertensive emergency, acute ischemic stroke (AIS) is currently one of the most common and severe neurological complications.^[3]^ Epidemiological studies have demonstrated that hypertension is the most important modifiable risk factor for all stroke subtypes, including ischemic stroke.^[4]^ Chronic hypertension has been shown to be closely associated with the development of cerebral small vessel disease and large artery atherosclerosis. During the acute phase, abrupt elevations in blood pressure exert stress on the vascular wall, which can lead to rupture of atherosclerotic plaques, subsequent thrombus formation, and arterial occlusion, thereby directly causing ischemic stroke. Moreover, in the context of severely elevated blood pressure, cerebral autoregulation may be impaired, resulting in cerebral edema and hypoperfusion, which further exacerbate ischemic injury to brain tissue.^[5,6]^

The coexistence of hypertensive emergency and AIS represents a major therapeutic challenge. Clinicians are required to achieve prompt blood pressure reduction to minimize further target-organ damage induced by acute hypertensive surges, while simultaneously avoiding an excessive decline that could impair cerebral perfusion and aggravate ischemic brain injury.^[7]^ This therapeutic paradox significantly complicates decision-making and underscores the importance of individualized management. In this context, we report 3 cases of hypertensive emergency complicated by AIS, outlining their clinical characteristics, underlying pathophysiological considerations, tailored therapeutic interventions, and the contribution of MDT collaboration. The purpose of this report is to offer practical reference for optimizing diagnostic and treatment strategies in similar clinical scenarios.

## 2. Case presentation

### 2.1. Case 1

**Figure 1.**
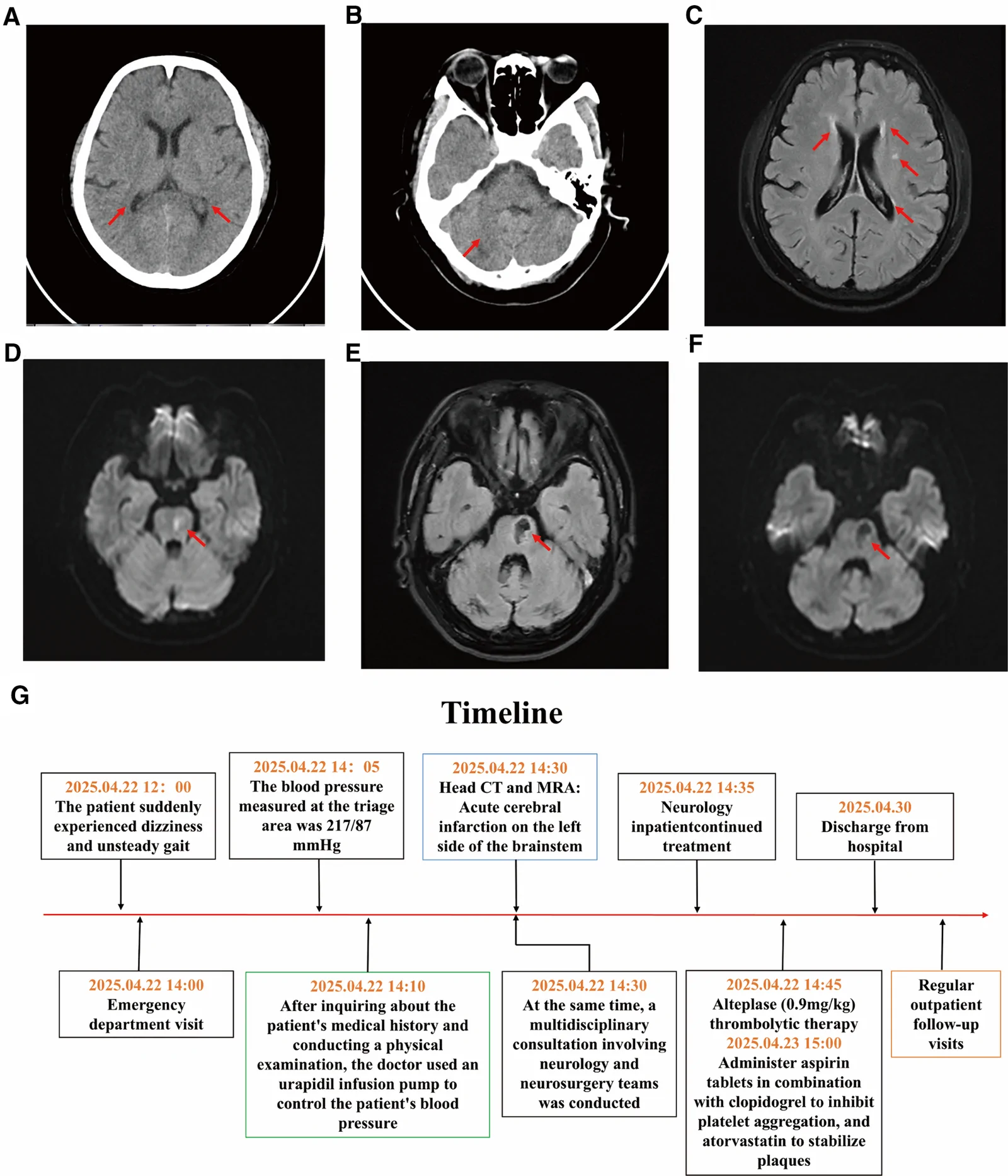
Cranial imaging of the patient. (A, B) CT scans demonstrate patchy hypodense regions in the right cerebellum and bilateral periventricular white matter, with poorly defined margins. (C, D) MRA images: small patchy hyperintense signals on DWI in the left brainstem; other sequences are inconclusive. Multiple small spot-like abnormal signals are observed in the bilateral centrum semiovale and periventricular white matter, with poorly defined borders, slightly hypointense on T1WI, hyperintense on T2WI/FLAIR, and no obvious signal change on DWI. (E, F) Follow-up images show multiple small spot-like abnormal signals in the left brainstem and bilateral centrum semiovale/periventricular white matter, with poorly defined borders, slightly hypointense on T1WI, and hyperintense on T2WI/FLAIR; patchy hypointense signals are seen in the left brainstem on DWI without obvious hyperintensity. (G) Timeline of events reported in this case. CT = computed tomography, DWI = diffusion-weighted imaging.

On April 22, 2025, a 61-year-old female patient was admitted with a sudden onset of dizziness lasting 2 hours. She had a history of hypertension and reported long-term use of amlodipine aspartate. Two hours prior to admission, she experienced dizziness and gait instability without obvious triggers. There were no complaints of urinary or fecal incontinence, loss of consciousness, or dysarthria. Her premorbid modified Rankin Scale (mRS) score was 1, while her National Institutes of Health Stroke Scale (NIHSS) score at admission was 0. At triage in the emergency department, the patient’s blood pressure was measured at 217/87 mm Hg, and she was immediately admitted to the emergency resuscitation area. Following rapid history taking and physical examination, the emergency physician initiated continuous intravenous urapidil via microinfusion pump, dynamically monitoring blood pressure and adjusting the infusion rate to gradually reduce systolic pressure to approximately 170 mm Hg within 1 hour. Laboratory investigations encompassing complete blood count, coagulation profile, homocysteine, blood glucose, inflammatory indices, and cardiac biomarkers were essentially within normal limits. However, lipid testing demonstrated slight elevations in low-density lipoprotein (LDL; 3.7 mmol/L) and total cholesterol (5.98 mmol/L). Non-contrast cranial CT demonstrated patchy hypodense areas in the right cerebellum and periventricular white matter bilaterally with indistinct margins (Fig. 1A, B). Further cranial MRA revealed a small patchy area of slightly hyperintense signal on diffusion-weighted imaging in the left brainstem, with other sequences inconclusive. Multiple small punctate abnormal signal lesions were also observed in the bilateral centrum semiovale and periventricular white matter, showing slightly low signal on T1WI, high signal on T2WI/FLAIR, and no apparent change on diffusion-weighted imaging, consistent with acute infarction in the left brainstem (Fig. 1C, D). Despite an initial NIHSS score of 0, MRI confirmed acute brainstem infarction consistent with a posterior circulation stroke. After multidisciplinary evaluation, intravenous thrombolysis was administered due to potential disabling neurological deterioration risk. Upon hospital admission, alteplase (rt-PA) was administered intravenously for thrombolysis, guided by the patient’s overall clinical assessment and supporting investigations. After 24 hours of stabilization, intravenous butylphthalide was introduced to promote collateral circulation and exert neuroprotective benefits. Secondary prevention strategies included initiation of aspirin plus clopidogrel for dual antiplatelet therapy and atorvastatin to stabilize atherosclerotic plaques. Antihypertensive therapy was optimized by transitioning to amlodipine besylate, facilitating stable blood pressure control, preservation of cerebral perfusion, and minimization of secondary vascular injury. Following this comprehensive management, the patient exhibited clinical improvement and was discharged with continued medical therapy. On follow-up on July 10, 2025, cranial MRA showed partial resolution of the previous infarct lesions with no evidence of disease progression (Fig. 1E, F). The patient’s neurological status remained stable with mild improvement compared with baseline, and no new ischemic lesions were observed during follow-up. The timeline of the patient’s diagnosis and treatment is shown in Figure 1G.

### 2.2. Case 2

**Figure 2.**
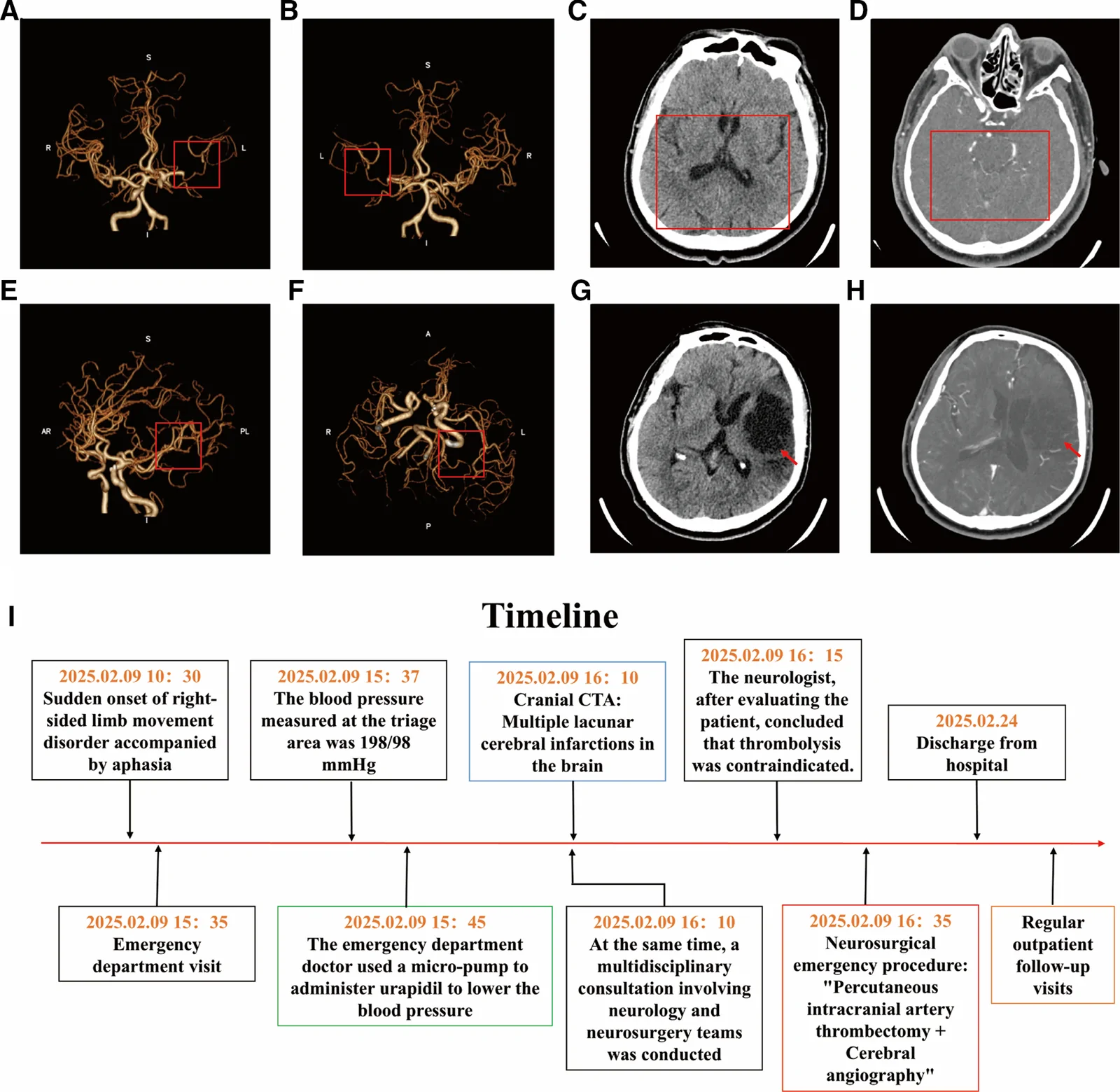
Cranial CTA images of the patient. (A, B) Occlusion of the terminal and distal segments of the left ICA; partial visualization of the intracranial segment of the left ICA; the distal M1 segment and branches of the left MCA are occluded. Mixed plaques with mild luminal stenosis are observed in the bilateral common carotid arteries, proximal external carotid arteries, and proximal ICAs. (C, D) Multiple small hypodense lesions of varying sizes with clear borders in the bilateral centrum semiovale and basal ganglia; multiple patchy hypodense areas with poorly defined borders in the bilateral centrum semiovale and periventricular white matter. (E, F) Calcified plaques in the walls of the left ICA at the C2–C4 segments with mild luminal stenosis; the C6 segment with moderate stenosis. Noncalcified plaques in the right ICA C6 segment and left posterior cerebral artery (P2) segment with mild luminal stenosis; the right anterior cerebral artery (A1) segment not visualized. (G–H) White matter lesions and formation of softening foci in the left frontal–insula–temporal region. (I) Timeline of events reported in this case. CTA = computed tomography angiography, ICA = internal carotid artery, MCA = middle cerebral artery.

On February 9, 2025, a middle-aged male patient was admitted with a sudden onset of right-sided limb weakness and aphasia lasting 5 hours. His medical history included prior cerebral infarction, hypertension, and diabetes mellitus. He also had a long-term history of smoking and alcohol consumption. Physical assessment demonstrated leftward ocular deviation, right facial droop, tongue deviation to the right, and a shallow right nasolabial fold. Motor testing indicated complete loss of strength in the right upper limb (0/5) and partial impairment in the right lower limb (2+/5). The right Babinski sign was elicited. His admission mRS score was 4, and upon presentation, his NIHSS score was 20. Upon admission to the emergency resuscitation area, his blood pressure was 198/98 mm Hg. After rapid history taking and physical examination, the emergency physician initiated continuous intravenous urapidil via microinfusion pump, adjusting the infusion rate to achieve a 15% to 25% reduction in blood pressure within 1 hour. Laboratory evaluation, including coagulation profile, homocysteine, and cardiac enzymes, was unremarkable. However, high-sensitivity C-reactive protein (CRP) was elevated at 24.46 mg/L, blood glucose measured 8.02 mmol/L, and lipid profile showed mild increases in LDL (3.44 mmol/L) and total cholesterol (5.47 mmol/L). Cranial computed tomography angiography (CTA) revealed occlusion of the distal segment and terminus of the left internal carotid artery, partial visualization of the intracranial segment of the left internal carotid artery (ICA), occlusion of the distal M1 segment and branches of the left middle cerebral artery (MCA), and mixed plaques in the proximal segments of the bilateral common, external, and internal carotid arteries with mild luminal stenosis (Fig. 2A, B). Multiple small hypodense lesions of variable size with clear boundaries were observed in the bilateral centrum semiovale and basal ganglia, and multiple patchy hypodense areas with indistinct margins were noted in the periventricular white matter bilaterally (Fig. 2C, D), consistent with multiple intracranial lacunar infarctions. Emergency consultations with neurology and neurosurgery were conducted. He had a history of cerebral infarction in January and had voluntarily discontinued antiplatelet therapy. Furthermore, the imaging results from this emergency visit indicated the presence of major vessel occlusion. Considering these combined factors, intravenous thrombolysis was contraindicated and was not expected to confer significant clinical benefit. The patient therefore underwent emergency endovascular thrombectomy combined with cerebral angiography. Postoperatively, vital signs remained stable. He was treated with enteric-coated aspirin for antiplatelet therapy and benazepril combined with amlodipine besylate to maintain SBP between 120 and 140 mm Hg. Following multidisciplinary consultation involving the ICU, neurology, neurosurgery, and rehabilitation teams, the patient was discharged to the rehabilitation department for continued therapy. During follow-up in the neurosurgery outpatient clinic, most recently on August 11, 2025, cranial CTA revealed calcified plaques with mild stenosis in the C2 to C4 segments and moderate stenosis in the C6 segment of the left internal carotid artery; noncalcified plaques with mild stenosis in the C6 segment of the right internal carotid artery and P2 segment of the left posterior cerebral artery; and non-visualization of the A1 segment of the right anterior cerebral artery (Fig. 2E, F). Brain white matter lesions and encephalomalacia in the left frontal, insular, and temporal lobes were also observed (Fig. 2G, H). A detailed timeline of the patient’s diagnostic and therapeutic course is presented in Figure 2I. At the most recent follow-up, the NIHSS score had improved to 10 and the mRS score was 2, indicating substantial functional recovery despite residual neurological deficits.

### 2.3. Case 3

**Figure 3.**
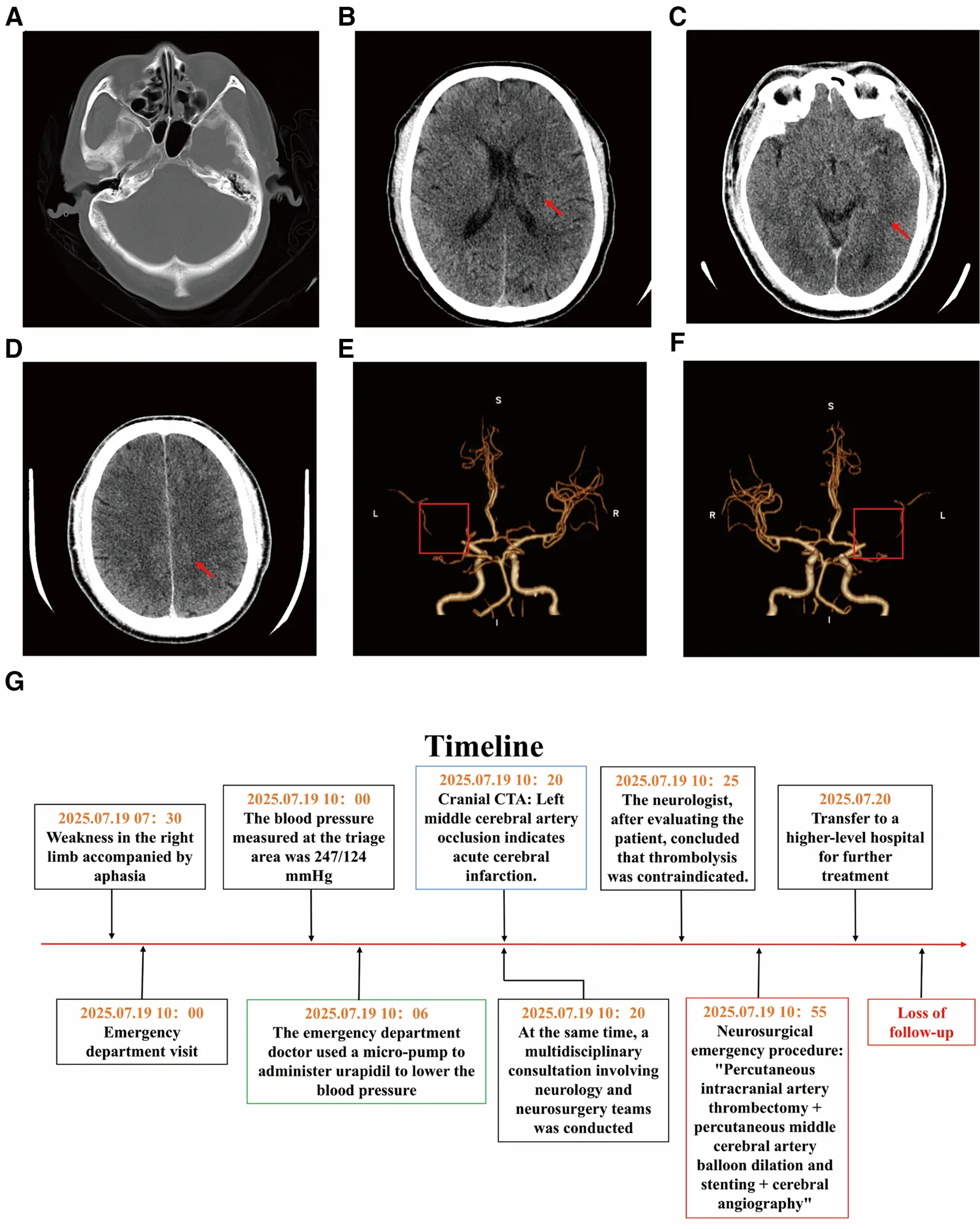
Cranial imaging of the patient. (A, B) CT images showing patchy hypodense areas with poorly defined borders in the left periventricular white matter; increased density is noted in the cerebellar tentorium and longitudinal fissure cistern. (C, D) CTA images showing absence of visualization of the left MCA; distal branch vessels appear sparse and faint. Calcified plaques are observed in the walls of the bilateral ICA. Other arteries, including bilateral anterior and posterior cerebral arteries and the right MCA, are clearly visualized, with normal course and no evidence of compression, displacement, stenosis, or occlusion. (E, F) Patchy hypodense areas with poorly defined borders in the left periventricular white matter. (G) Timeline of events reported in this case. CT = computed tomography, CTA = computed tomography angiography, ICA = internal carotid artery, MCA = middle cerebral artery, MDT = multidisciplinary team, mRS = modified Rankin Scale, NIHSS = National Institutes of Health Stroke Scale.

On July 19, 2025, a 46-year-old male patient was admitted after his son reported that, 2.5 hours prior, the patient developed sudden right-sided limb weakness and aphasia without obvious triggers. His medical history included hypertension and diabetes mellitus, with irregular adherence to antihypertensive medications. On admission, his blood pressure was 247/124 mm Hg, and continuous intravenous urapidil via microinfusion pump was initiated in the emergency department for blood pressure control. Physical examination revealed rightward tongue deviation, shallow right nasolabial fold, sluggish pupillary light reflexes bilaterally, right-sided limb weakness, preserved left-sided limb movement, no edema in the lower extremities, positive right Babinski sign, and negative left Babinski sign. The mRS score was 4 and the NIHSS score was 15. Laboratory tests including coagulation profile, homocysteine, cardiac enzymes, and LDL were unremarkable, except for elevated high-sensitivity CRP at 10.09 mg/L and blood glucose of 7.42 mmol/L. Non-contrast cranial CT demonstrated patchy hypodense areas in the periventricular white matter of the left lateral ventricle with indistinct margins, and slightly increased density in the cerebellar tentorium and longitudinal fissure cistern (Fig. 3A, B). Further cranial CTA revealed low-density lesions in the left periventricular white matter consistent with infarction, as well as occlusion of the left middle cerebral artery, indicative of acute cerebral infarction (Fig. 3C–F). Due to the extensive occlusion of large blood vessels and the need for immediate endovascular recanalization treatment, the patient underwent emergency neurosurgical intervention, including percutaneous intracranial arterial thrombectomy, balloon angioplasty of the middle cerebral artery, cerebral angiography, femoral artery puncture, and femoral artery closure. Postoperatively, blood pressure was continuously managed with urapidil via microinfusion pump, and antiplatelet therapy with tirofiban and statins were administered to stabilize atherosclerotic plaques and reduce the risk of recurrent stroke. Unfortunately, the patient’s family decided to transfer him to a higher-level hospital for further treatment, resulting in the loss of follow-up. The timeline of the patient’s diagnosis and treatment is shown in Figure 3G.

A summary of the baseline characteristics, imaging findings, treatment strategies, and outcomes of the 3 patients is provided in Table 1.

**Table 1:** Summary of clinical characteristics, management, and outcomes of the 3 cases.

Variable	Case 1	Case 2	Case 3
Age/sex	61/F	Middle-aged/M	46/M
Medical history	Hypertension	Hypertension Diabetes Prior stroke	Hypertension Diabetes
Initial BP (mm Hg)	217/87	198/98	247/124
NIHSS	0	20	15
mRS	1	4	4
Imaging findings	Brainstem infarction	Left ICA/MCA occlusion	Left MCA occlusion
Reperfusion therapy	Intravenous thrombolysis	Mechanical thrombectomy	Mechanical thrombectomy
BP management	Intravenous urapidil	Intravenous urapidil	Intravenous urapidil
Follow-up outcome	Neurological status stable with mild improvement; no recurrent ischemic lesions	NIHSS 10; mRS 2	Lost to follow-up

BP = blood pressure, ICA = internal carotid artery, MCA = middle cerebral artery, mRS = modified Rankin Scale, NIHSS = National Institutes of Health Stroke Scale.

## 3. Discussion

According to the 2017 American College of Cardiology/American Heart Association (ACC/AHA) Clinical Practice Guidelines,^[8]^ a hypertensive emergency is defined as a SBP >180 mm Hg and/or diastolic blood pressure >120 mmHg, accompanied by new or progressively worsening target-organ damage mediated by the acute rise in blood pressure. It is characterized by a sudden and severe elevation in blood pressure, accompanied by vascular injury to multiple target organs, including the retina, brain, and kidneys.^[9]^ AIS is an emergency condition caused by interrupted cerebral blood flow leading to brain tissue injury. Clinically, it often presents with sudden onset of limb weakness, facial palsy, headache or dizziness, and speech or comprehension difficulties, and may be preceded by transient ischemic attack (TIA) symptoms.^[10]^ Hypertensive emergency complicated by AIS represents a critically severe condition, frequently occurring in middle-aged and elderly patients with long-standing poorly controlled hypertension.^[11]^ Studies have shown that among patients with hypertensive emergency, the incidence of AIS is significantly higher than that of other complications, such as acute myocardial infarction or aortic dissection. However, acute blood pressure management in patients with hypertensive emergency complicated by AIS remains a clinical dilemma. Excessively aggressive blood pressure reduction may lead to inadequate cerebral perfusion and expansion of the ischemic penumbra, whereas uncontrolled hypertension increases the risk of hemorrhagic transformation and cerebral edema.^[12]^ Therefore, adherence to stepwise, individualized, and guideline-recommended blood pressure targets remains fundamental in the management of hypertensive emergencies. This report details 3 cases, focusing on their imaging profiles, evidence-based antihypertensive strategies, tailored therapeutic approaches, and coordinated multidisciplinary care, aiming to inform clinicians on best practices for similar patients and improve outcomes.

Hypertensive emergency is not only a risk factor for AIS but also a direct precipitating event. In the 3 cases reported here, all patients exhibited a sudden rise in blood pressure at stroke onset, primarily through 2 key mechanisms. First, plaque rupture and thrombosis: poorly controlled long-standing hypertension promotes the formation of atherosclerotic plaques within the arterial wall. A subsequent abrupt elevation in blood pressure exerts excessive mechanical stress on the vessel wall, leading to plaque rupture (as observed in case 2, where CTA revealed multiple plaques and stenoses in the internal carotid and middle cerebral arteries). Exposure of collagen and tissue factor from the ruptured plaque rapidly activates platelets and the coagulation cascade, resulting in local thrombus formation and artery-to-artery embolism, ultimately occluding distal cerebral vessels and causing AIS.^[13]^ In case 3, the observed acute occlusion of the middle cerebral artery was likely caused by this mechanism. Second, cerebral perfusion abnormalities: the brain possesses intrinsic autoregulatory mechanisms that maintain relatively stable cerebral blood flow within a certain blood pressure range. However, in patients with chronic hypertension, this autoregulatory capacity is impaired, requiring higher blood pressure to sustain adequate cerebral perfusion. During a hypertensive emergency, autoregulation may fail completely. Such failure can lead to 2 consequences: on 1 hand, hyperperfusion may occur, resulting in hypertensive encephalopathy, cerebral edema, or hemorrhagic transformation; on the other hand, in distal regions of stenotic arteries, turbulent flow and endothelial dysfunction may paradoxically cause insufficient cerebral perfusion, further exacerbating ischemic injury to brain tissue.^[14]^ Although the patient in case 1 did not exhibit severe neurological deficits, imaging revealed white matter lesions – markers of chronic cerebral hypoperfusion and blood–brain barrier disruption caused by long-standing hypertension – reflecting this mechanism. In summary, hypertensive emergency contributes to the onset and progression of acute ischemic stroke through a combined effect of hemodynamic disturbances and vascular structural injury. Importantly, despite well-established guideline frameworks for blood pressure management in acute ischemic stroke, several practical uncertainties remain unresolved in real-world emergency settings. Current recommendations primarily define target blood pressure thresholds and general pharmacologic options; however, they provide limited operational guidance regarding drug selection under rapidly evolving clinical scenarios where stroke subtype confirmation, reperfusion eligibility, and neurological severity assessment occur concurrently. In this context, our case series highlights a clinically relevant gap between guideline-based targets and bedside implementation, particularly in the early phase of hypertensive emergency complicated by suspected or confirmed AIS. The present cases therefore provide incremental evidence regarding real-time therapeutic decision-making, emphasizing how individualized hemodynamic control strategies can be operationalized in routine emergency workflows.

**Figure 4.**
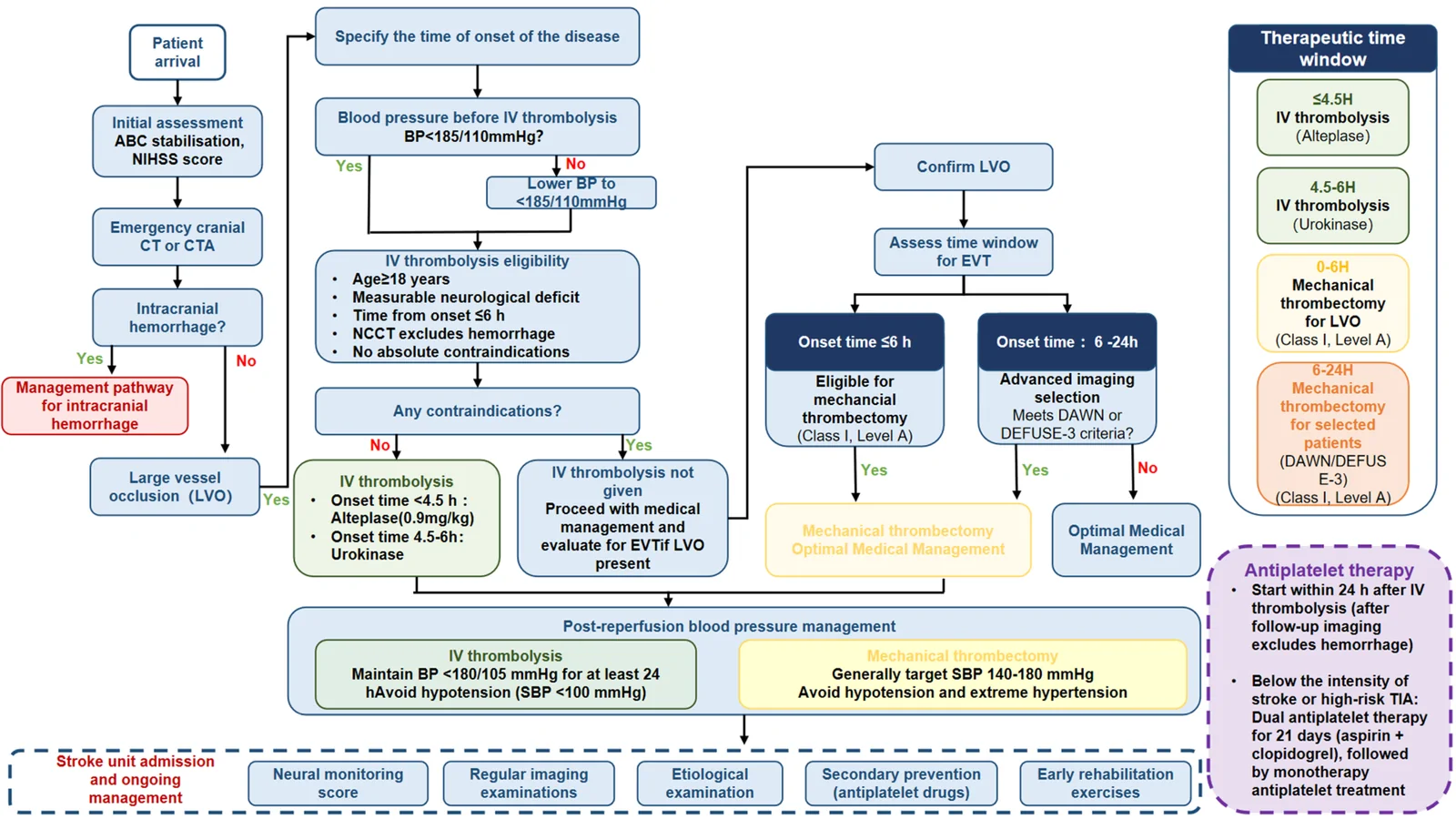
Clinical decision-making flowchart for intravenous thrombolysis and mechanical thrombectomy in acute ischemic stroke. ABC = airway/breathing/circulation, BP = blood pressure, CT = computed tomography, CTA = computed tomography angiography, EVT = endovascular thrombectomy, IV = intravenous, LVO = large vessel occlusion, NCCT = non-contrast computed tomography, NIHSS = National Institutes of Health Stroke Scale, SBP = systolic blood pressure, TIA = transient ischemic attack.

The 3 cases reported herein highlight the clinical management challenges of AIS complicated by hypertensive emergency. According to the AHA/ASA guidelines, for patients eligible for intravenous thrombolysis or mechanical thrombectomy, blood pressure must be reduced to <180/110 mm Hg prior to the procedure to meet treatment eligibility criteria. Post-thrombectomy, blood pressure is generally recommended to be maintained at <180/105 mm Hg, whereas for patients with successful recanalization, lower targets (e.g., SBP <140 mm Hg) may be appropriate to reduce the risk of reperfusion injury and intracerebral hemorrhage.^[15]^ Recent major international guidelines – including the 2025 AHA, 2024 ESC, 2023 ESH, and 2025 JSH guidelines – demonstrate strong convergence on blood pressure thresholds for reperfusion therapy. For patients receiving intravenous thrombolysis, all consistently recommend lowering blood pressure to <185/110 mm Hg before treatment and maintaining <180/105 mm Hg during the first 24 hours. For those undergoing endovascular thrombectomy (EVT), it is reasonable to maintain blood pressure at ≤180/105 mm Hg during and for 24 hours after the procedure.^[16]^ However, recent randomized controlled trials have challenged the assumption that “lower is always better” after successful reperfusion. The ENCHANTED-2 MT, OPTIMAL-BP, and BEST-II trials have shown that pushing SBP below 140 mm Hg after successful EVT may worsen outcomes, leading to a class 3, level A recommendation against overly aggressive post-reperfusion blood pressure lowering.^[17-19]^ Current consensus favors individualized SBP targets below 160 mm Hg after reperfusion, with higher targets (up to 180 mm Hg) considered in cases of incomplete reperfusion, and excessively low targets (<120 mm Hg) deemed potentially detrimental. Several large randomized controlled trials have examined prehospital blood pressure lowering in the hyperacute setting. The INTERACT4 trial – the largest prehospital blood pressure management study to date – randomized 2404 patients with suspected stroke and elevated blood pressure to receive intensive blood pressure lowering (target SBP 130–140 mm Hg) with urapidil versus usual care in the ambulance. The trial was neutral overall but revealed a critical interaction: patients with hemorrhagic stroke benefited from intensive blood pressure lowering, whereas those with ischemic stroke had worse functional outcomes (common OR 1.30, 95% CI: 1.06–1.60).^[20]^ The RIGHT-2 and MR ASAP trials similarly failed to demonstrate overall benefit from early blood pressure control and raised safety concerns in ischemic stroke patients.^[21,22]^ These findings underscore that aggressive prehospital blood pressure reduction in undifferentiated stroke may be harmful in AIS patients by compromising perfusion to the ischemic penumbra before stroke subtype is confirmed. The TRUTH study compared active blood pressure lowering to achieve <185/110 mm Hg before intravenous thrombolysis versus waiting for spontaneous reduction, finding a trend toward worse functional outcomes with active lowering (adjusted OR 1.27, 95% CI: 0.96–1.68) despite shorter door-to-needle times.^[23]^ This observation has prompted the ongoing BALANCE trial, which is comparing concurrent (simultaneous thrombolysis and blood pressure lowering) versus sequential (blood pressure lowering first) management strategies in AIS patients with elevated blood pressure.^[16]^ In all 3 cases, the emergency physicians implemented an active yet controlled blood pressure reduction strategy, achieving a 15% to 25% decrease within 1 hour. This approach is fully consistent with guideline recommendations emphasizing cautious and gradual blood pressure lowering to avoid abrupt drops, effectively protecting the ischemic penumbra in the brain. In patients with hypertensive emergency complicated by acute ischemic stroke, selection of an intravenous antihypertensive agent should be guided not only by guideline recommendations but also by the patient’s hemodynamic status, anticipated reperfusion therapy, and institutional workflow. Although both nicardipine and labetalol are recommended by contemporary international guidelines, each has context-specific limitations. Nicardipine provides predictable blood pressure control but may induce reflex sympathetic activation and tachycardia due to peripheral vasodilation,^[24]^ whereas labetalol, despite its established efficacy, may be less suitable in patients with impaired cardiac function because of its combined α- and β-adrenergic blockade. In the present series, intravenous urapidil was selected for early blood pressure management because its pharmacological profile was considered well-suited to the clinical scenario. Urapidil combines peripheral selective α1-adrenergic receptor antagonism with central 5-hydroxytryptamine receptor 1A (5-HT1A) agonist activity, allowing gradual blood pressure reduction while attenuating reflex sympathetic activation.^[25]^ Experimental and clinical studies have shown that urapidil effectively lowers blood pressure without significantly increasing intracranial pressure and may better preserve cerebral perfusion during acute blood pressure reduction.^[26]^ Continuous intravenous infusion also permits rapid dose titration, enabling clinicians to achieve guideline-recommended blood pressure targets while minimizing abrupt hemodynamic fluctuations that could potentially compromise the ischemic penumbra. In our 3 patients, this titratable strategy facilitated timely blood pressure stabilization before reperfusion therapy without clinically significant hypotension or treatment delay, suggesting that urapidil represents a practical option for individualized blood pressure management in this setting, particularly where continuous bedside monitoring is available. The 2023 Chinese multidisciplinary expert consensus recommends urapidil as a first-line agent for blood pressure control in AIS patients undergoing intravenous thrombolysis.^[27]^ However, in clinical practice, the selection of antihypertensive drugs requires comprehensive consideration of multiple factors. First, urapidil has a relatively short half-life and requires continuous intravenous infusion to maintain efficacy. Therefore, it places high demands on infusion pump equipment and nursing monitoring capabilities and may encounter certain implementation difficulties in resource-limited medical environments. Second, although urapidil is widely used in some countries, its accessibility and approval status vary globally, which to a certain extent limits its widespread application as a first-line drug. Third, in patients with myocardial ischemia or aortic dissection risk, labetalol is preferred over urapidil in clinical practice due to its dual α- and β-receptor blocking effects.^[28]^ While nicardipine has a predictable dose–response relationship and stable blood pressure control, it has relatively mature application experience in neuro-intensive care, and thus remains a widely used alternative option.^[29]^ In this report, 3 patients all received urapidil for blood pressure management due to hospital resource constraints. However, the overall results showed good efficacy and safety. Currently, there are many options for intravenous blood pressure-lowering drugs. In clinical practice, individualized decisions should be made based on the patient’s individual condition, coexisting diseases, and medical conditions. More high-quality studies are still needed to clarify the optimal blood pressure-lowering strategy in complex clinical situations. The indications for thrombolysis and mechanical thrombectomy are critical in the management of acute stroke (Fig. 4). Reperfusion decisions were individualized based on imaging findings, stroke severity, and contraindications to thrombolysis, in accordance with the AHA/ASA and ESO guidelines, which emphasize that treatment decisions should not rely solely on NIHSS scores but should integrate clinical presentation and imaging features.^[30,31]^ According to current guideline recommendations, intravenous thrombolysis with recombinant tissue plasminogen activator (rt-PA, 0.9 mg/kg) remains the first-line therapy within 4.5 hours of stroke onset. Between 4.5 and 6 hours, urokinase may be considered as an alternative agent. For patients with contraindications to intravenous thrombolysis, mechanical thrombectomy is the preferred strategy to restore cerebral perfusion. In cases where neither thrombolysis nor thrombectomy is feasible, dual antiplatelet therapy with aspirin and clopidogrel, in combination with atorvastatin for plaque stabilization, is advised.^[32]^ Within this reperfusion treatment framework, the therapeutic benefit must be carefully weighed against the potential risks, underscoring the importance of rigorous eligibility assessment and accurate imaging evaluation. In patients with concomitant hypertensive emergency, balancing blood pressure control with the therapeutic time window represents a key clinical challenge. Cases 2 and 3 did not undergo intravenous thrombolysis due to contraindications, whereas case 1 illustrates a common clinical dilemma: when a patient is within the thrombolytic time window and exhibits significant neurological deficits, blood pressure must be rapidly yet safely reduced to <185/110 mm Hg. In this context, urapidil administered via microinfusion pump offers a unique advantage due to its rapid onset and high controllability. This management process is essentially a race against time, requiring precise balancing between the benefits of reperfusion and the risk of hypoperfusion from excessive blood pressure reduction. Successful execution heavily depends on meticulous dynamic blood pressure monitoring by nursing staff in conjunction with the physician’s clinical expertise.^[33]^ It is noteworthy that in cases 2 and 3, patients developed hypertensive emergencies and subsequent strokes due to long-term discontinuation or irregular use of antihypertensive medications, resulting in chronically uncontrolled blood pressure. This highlights the insidious and harmful nature of hypertension, often referred to as the “silent killer,” and underscores the critical importance of regular medication adherence, long-term blood pressure monitoring, and lifelong healthy lifestyle interventions. In summary, these 3 cases highlight that the management of hypertensive emergency complicated by acute ischemic stroke extends beyond achieving guideline-recommended blood pressure targets and requires individualized, dynamic hemodynamic management integrated with reperfusion decision-making. Our experience suggests that continuous intravenous urapidil infusion may represent a feasible option for controlled blood pressure reduction in appropriately selected patients, facilitating timely reperfusion while minimizing abrupt hemodynamic fluctuations. Rather than supporting the superiority of a specific antihypertensive agent, these cases emphasize that drug selection should be individualized according to pharmacological characteristics, patient condition, treatment strategy, and institutional resources. Collectively, these observations provide practical insights into translating guideline recommendations into real-world emergency practice and underscore the importance of coordinated multidisciplinary management in optimizing patient outcomes.

This case series has several limitations, including a small sample size from a single center and the absence of a control group, which limits the generalizability of the findings. In addition, the observational design precludes causal inference. A further limitation is that long-term follow-up data were unavailable for Case 3 because the patient was transferred to another institution shortly after treatment. Consequently, long-term outcomes can only be confirmed for Cases 1 and 2, and the overall prognostic findings should be interpreted with caution.

## 4. Conclusion

Hypertensive emergency complicated by acute ischemic stroke (AIS) poses a frequent yet complex clinical challenge. Effective management requires balancing blood pressure control with the preservation of adequate cerebral perfusion. Individualized therapeutic plans should account for the patient’s hemodynamic status, disease severity, and cerebral perfusion needs, with careful monitoring serving as the cornerstone for safe blood pressure reduction. While precise management helps minimize stroke-related complications, overly rapid lowering may be harmful. MDT involvement is essential for optimizing treatment outcomes and enhancing long-term quality of life.

## Author contributions

**Conceptualization:** Zhouyang Gu.

**Data curation:** Don Zhu, Xiaoyu Liu, Xiaoyang Zhang.

**Methodology:** Xiaoyu Liu.

**Funding acquisition:** Keyuan Zhao.

**Writing – original draft:** Weihao Wang.

**Writing – review & editing:** Zhouyang Gu.
